# Searchable database of frequent R-groups in medicinal chemistry and their preferred replacements

**DOI:** 10.1016/j.dib.2021.107456

**Published:** 2021-10-08

**Authors:** Kosuke Takeuchi, Ryo Kunimoto, Jürgen Bajorath

**Affiliations:** Department of Life Science Informatics, B-IT, LIMES Program Unit Chemical Biology and Medicinal Chemistry, Rheinische Friedrich-Wilhelms-Universität, Friedrich-Hirzebruch-Allee 6, Bonn D-53115, Germany

**Keywords:** Medicinal chemistry, Compound optimization, Analog series, R-groups, Network analysis, R-group replacement database

## Abstract

In compound optimization, analogue series (ASs) are generated by introducing different R-groups (substituents, functional groups) at specific substitution sites. Systematic investigations of R-groups in medicinal chemistry have so far been rare. We have carried out a large-scale computational analysis of R-groups on the basis of ASs covering currently available bioactive compounds (Takeuchi et al., 2021). With the aid of a network data structure, frequently used R-groups and preferred replacements were identified. On the basis of these data, R-group replacement hierarchies were derived and organized in a searchable database that is made freely available. This contribution complements our systematic analysis (Takeuchi et al., 2021) by specifying the data we have generated and detailing their open access deposition.

**Specifications Table**

**Table:** 

Subject	Medicinal chemistry, drug discovery
Specific subject area	Computational analysis of active compounds, extraction of R-groups from analogue series, identification of frequently used R-groups and preferred replacements, design of a database system.
Type of data	Tables Text files (CSV, XML)
How data were acquired	Qualifying active compounds were systematically curated from ChEMBL [2]. All R-group data were generated in-house.
Data format	Secondary data Tables in text files (consistently formatted, searchable)
Parameters for data collection	1. Compound data from ChEMBL: 1.1 High-confidence activity annotations for single target proteins. 2. In-house generated R-group data: 2.1. Identification of analogue series with at least three compounds, 2.2. Substitution site-specific sampling of R-groups and replacements, 2.3. Network-based identification of frequently used R-groups and preferred replacements.
Description of data collection	The network data structure for R-group replacements is made available as a CSV file. For the 500 most frequently used R-groups, systematically generated replacement sequences are provided in a searchable XML file. R-group structures are reported as SMILES strings [3].
Data source location	Department of Life Science Informatics and Data Science, B-IT, University of Bonn, Friedrich-Hirzebruch-Allee 6, D-53115 Bonn, Germany.
Data accessibility	The data sets are freely available in a deposition on the Zenodo open access platform that is accessible via the following link: https://doi.org/10.5281/zenodo.4741973. In addition, the data sets are deposited on Mendeley Data and can be accessed via the following link: https://doi.org/10.17632/pz7kkhk2bv.1.
Related research article	K. Takeuchi, R. Kunimoto, J. Bajorath, Systematic Mapping of R-group Space Enables the Generation of an R-group Replacement System for Medicinal Chemistry, Eur. J. Med. Chem. 2021, 225, 113771. https://doi.org/10.1016/j.ejmech.2021.113771[1].

**Value of the Data**

• The collection of frequently used R-groups and associated replacement hierarchies provides a large knowledge base for compound optimization. R-groups of interest can be searched using the new database, the first of its kind, and suitable replacements across bioactive compounds can be identified, hence providing practical decision support for medicinal chemistry which analogues to consider next.
• Since the computational approach for R-group identification was generalized (see below), we have obtained the -to our knowledge- by far largest collection of formally defined R-groups available thus far, with more than 50,000 candidate groups.
• In addition to the R-group replacement database, we also provide the original network data structure from which frequent R-groups and their replacements were obtained. Thus, the R-group database and replacment system can be modified or extended according to user preferences.

## 1 Data Description

From 343,373 qualifying bioactive compounds obtained from ChEMBL [2], a total 17,254 ASs with at least three analogues (comprising 314,525 unique compounds) were extracted using an algorithmic variant [1] of the compound-core relationship (CCR) method [4]. From each AS, the core structure was isolated and substitution sites were indexed, yielding a total of 61,312 sites. For each substitution site, all R-groups from the corresponding AS were sampled, leading to the identification of a total of 50,545 unique R-groups. For each site, all possible pairwise R-group replacements were enumerated. Then, replacements for all 61,312 substitution sites were combined.

On the basis of these data, a global R-group network was constructed in which nodes represented individual R-groups and edges pairwise replacements. Two network parameters were calculated including the ‘node degree’ (ND; defined as the number of replacements/edges per node) and the ‘edge weight’ (EW; defined as the frequency with which each unique replacement occurred over all substitution sites). Most frequently used R-groups were identified on the basis of ND values and their preferred replacements on the basis of EW values.

The initially obtained global network structure was analyzed and many replacements of frequent R-groups were found to be dominated by the H atom, methyl group, and phenyl ring. Among these, the H atom was a special case because it was always present at a substitution site before a replacement with another R-group occurred and involved in a replacement when another group was introduced. To avoid domination of preferred replacements by these three groups, we further refined the network structure by omitting them, yielding the final network containing a total of 50,311 unique R-groups and 2,937,991 pairwise replacements.

The final network was used to identify preferred replacements of frequent R-groups having largest EW values. Therefore, we determined ‘first layer’ and ‘second layer’ R-group replacements. First layer replacements were formed by immediate network neighbors of a prioritized R-group and second layer replacements by nearest neighbors of the first layer groups. We then systematically generated ‘5 × 2’ first and second layer replacements. Hence, for a selected R-group, this data structure combined the top five most frequent first layer replacements with the top two second layer replacements (if available).

**Fig. 1:**
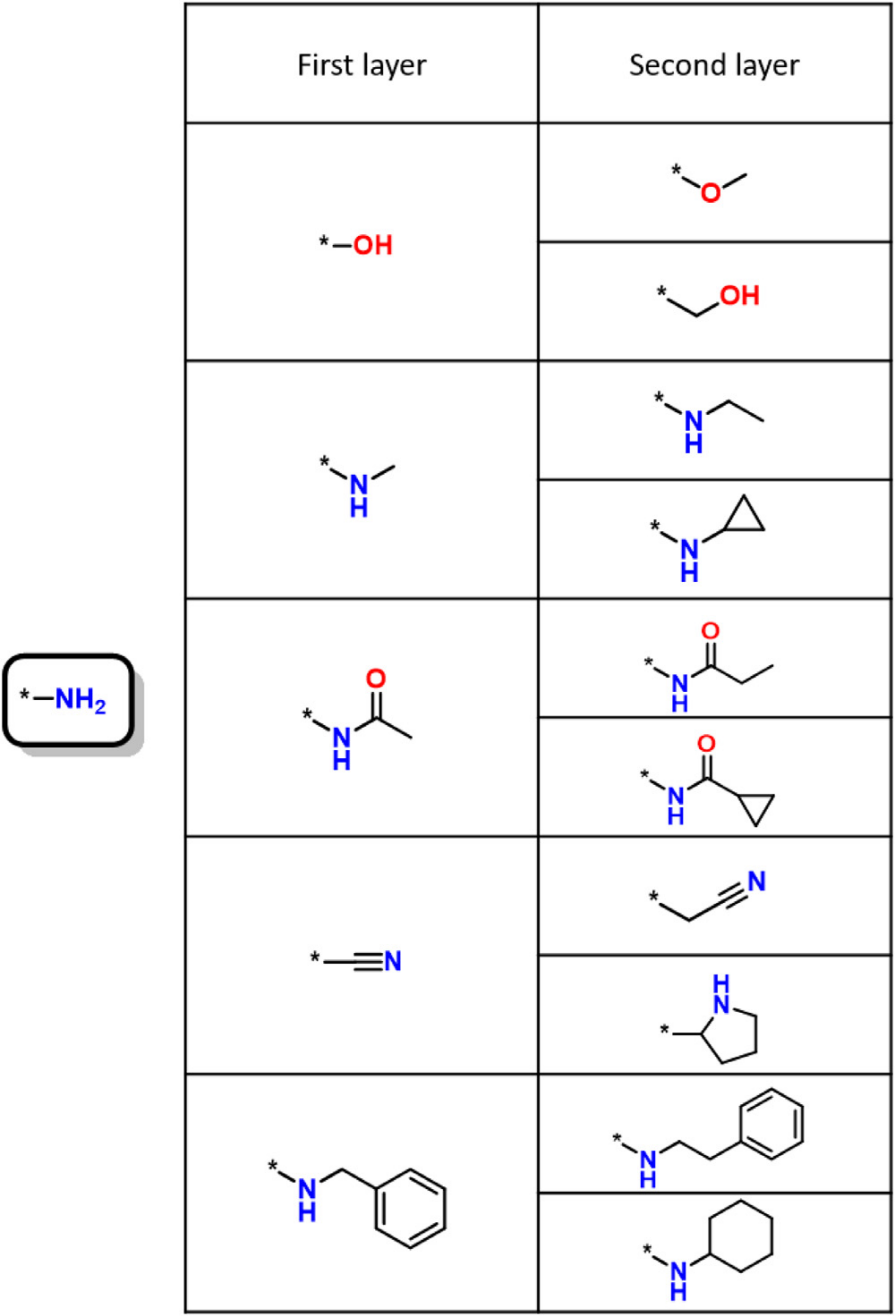
Exemplary R-group replacement hierarchy. Shown is the 5 × 2 replacement hierarchy for the amino group (left), one of the most frequent R-groups. Standard atom coloring is used.

Fig. 1 shows an exemplary 5 × 2 replacement hierarchy. For a given R-group, this data structure formally defines 10 first and second layer replacement sequences.

The 500 most frequently used R-groups were determined and for each of these groups, the 5 × 2 replacement hierarchy was generated.

On the basis of the network data, the composition of replacement hierarchies can be modified at will. Since the dominant methyl group and phenyl ring were omitted from the final network (in addition to the H atom), these R-groups can be considered as additional generic replacements in all 5 × 2 hierarchies.

The 50,311 unique R-groups and 2,937,991 pairwise replacements forming the final network are provided as a CSV file containing three columns (source, target, and weight) defining weighted edges and participating nodes. In addition, the database containing the 500 most frequently used R-groups from the final network and the 5 × 2 replacement hierarchies is made available as a searchable XML file. Both files are contained in our open access depositions specified above.

## 2 Experimental Design, Materials and Methods

The analysis involved the following steps: (1) Compound selection; (2) Systematic identification of ASs; (3) Core structure isolation and indexing of substitution sites; (4) Site-specific sampling of R-groups; (5) Generation of an R-group network data structure revealing frequent R-groups and replacements; and (6) Design of R-group replacement hierarchies. All compounds and ASs were recorded and processed as SMILES strings [3]. The CCR method [4] systematically decomposes compounds and generates ASs with indexed substitution sites. For our analysis, the approach was generalized such that decomposition based on pre-defined chemical rules was replaced by systematic fragmentation of exocyclic single bonds, thereby substantially increasing fragment space [1]. Steps (4) and (6) were carried out with in-house scripts. The network data structure was generated and analyzed using Cytoscape [5].

## Ethics Statement

This is a secondary data set and thus did not involve any human or animal testing.

## CRediT authorship contribution statement

**Kosuke Takeuchi:** Data curation, Formal analysis, Writing – review & editing. **Ryo Kunimoto:** Conceptualization, Formal analysis, Writing – review & editing. **Jürgen Bajorath:** Conceptualization, Supervision, Formal analysis, Writing – original draft, Writing – review & editing.

## Declaration of Competing Interest

The authors declare that they have no known competing financial interests or personal relationships which have, or could be perceived to have, influenced the work reported in this article.
